# Dietary food additive monosodium glutamate with or without high-lipid diet induces spleen anomaly: A mechanistic approach on rat model

**DOI:** 10.1515/biol-2022-0004

**Published:** 2022-01-29

**Authors:** Debasmita Das, Arnab Banerjee, Ankita Bhattacharjee, Sandip Mukherjee, Bithin Kumar Maji

**Affiliations:** Department of Physiology (UG & PG), Serampore College, 9 William Carey Road, Serampore, Hooghly-712201, West Bengal, India

**Keywords:** food additives, monosodium glutamate, high-lipid diet, spleen, oxidative stress

## Abstract

Globally, the trend of using food additives and eating ready-made fast food has led to a deleterious impact on immune organs. Monosodium glutamate (MSG), as a food additive in a high-lipid diet (HLD), acts as a silent killer of immune cells. Hence, the present study aimed to evaluate the role of MSG in HLD on spleen injury in rats. Results showed that a 2.52-fold and 1.91-fold increase in spleen index in MSG and MSG + HLD group indicates splenomegaly, whereas a 1.36-fold and 1.29-fold increase in pro-inflammatory cytokines in MSG and MSG + HLD-fed rats, respectively, promote the inflammatory response. Additionally, MSG and MSG + HLD induce oxidative stress by 1.81-fold and 1.1-fold increased generation of reactive oxygen species (ROS) in macrophage population, and 1.38-fold and 1.36-fold increased generation of ROS in lymphocytes population, respectively. Furthermore, mitochondrial membrane potential was significantly reduced by 1.43-fold and 1.18-fold in MSG and MSG + HLD groups. Therefore, the current study argues that MSG has more detrimental effects on the spleen than MSG + HLD due to the presence of antioxidants in HLD, which suppresses the deleterious impact of MSG. Hence, it can be inferred that MSG induces spleen injury via targeting redox-guided cellular signaling with inflammatory response, leading to severe immune system anomalies.

## Graphical abstract

**Figure:**
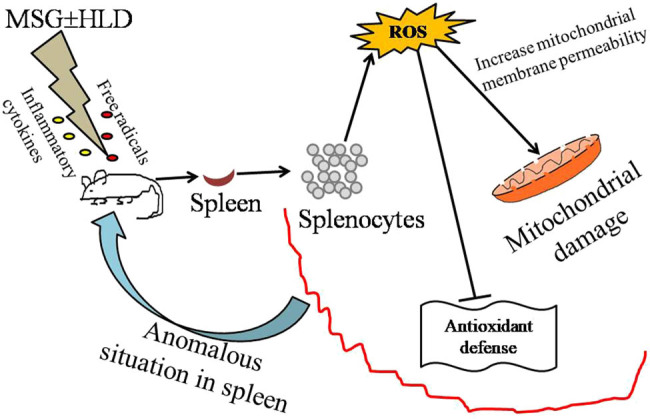

## 1 Introduction

Eating habits are rapidly changing worldwide, and a dependency on ready-made fast food with minimal physical activity leads to health problems [1,2]. In most ready-made fast-food, saturated fats such as palmitic acid, lauric acid, and myristic acid are abundant along with hydrogenated fats and trans fatty acids, resulting in oxidative stress, which can cause inflammation, apoptosis, metabolic disorders, and systemic inflammatory diseases [3,4,5,6]. Moreover, using widely used food additive, monosodium glutamate (MSG), in ready-made fast food enhances the palatability of the meals, but significantly affects the appetite center, and thereby results in overweight and different adverse impacts on humans and experimental animals [7,8]. MSG is suspected of increasing the generation of free radicals in the body, which leads to a decrease in the body’s antioxidant production. This results in oxidative damage throughout the body [1,2,4]. On the other hand, the health and function of the immune system are closely related to nutritional status [9]. A malnourished individual is more vulnerable to infections and immune dysfunction. Currently, malnutrition refers to inadequate nutrition as well as nutritional imbalances, such as excessive energy intake and obesity. The adverse effects of obesity include impaired immune function, which is reflected in an inflammatory response that is not resolved, and impaired immune surveillance [10].

In addition, obesity is associated with oxidative stress, either because antioxidant defenses are weakened or oxidants are formed in excess. A detrimental feedback loop can be closed by oxidative stress, damaging cellular structures and triggering an inflammatory response [2,4,10]. Although there have been several studies focusing on the obesity and immunity research on circulating leukocytes, on the other hand, the immune system itself is also affected [10]. However, very few reports are available on the impact of MSG or flavor-enhancing high-lipid diet (HLD) in the spleen, which is directly linked with overweight and systemic low-grade inflammation. Consequently, it is important to study the adverse impact of MSG with or without HLD as a component of ready-made fast food on the spleen, the largest secondary lymphoid organ, and the site of mechanical filtration of red blood cells in the body. Moreover, the spleen tissue is vulnerable to oxidative stress induced by different xenobiotics, contributing to apoptosis in splenocytes [11]. Reactive oxygen species (ROS) are a highly reactive form of oxygen that damages proteins, nucleic acids, lipids, membranes, and organelles such as mitochondria, which activates cell death processes, including apoptosis [2,4,11]. Mitochondrial dysfunction, death receptors, and the endoplasmic reticulum mainly regulate apoptosis during oxidative stress [11]. In addition, the spleen is responsible for destroying spent blood cells, antigens, and other foreign compounds [12,13]. Therefore, the immunotoxic effects of MSG alone or in combination with HLD on B and T lymphocytes may be manifested in the spleen due to the presence of these cell types. Hence, the specific aim of the present study is to assess the immunotoxic effect of MSG with or without HLD on the spleen by using a male rat model, and to elucidate the possible mechanism for this immunotoxic effect, if any.

## 2 Materials and methods

### 2.1 Chemicals

MSG (SRL, India), Thiobarbituric acid (TBA), 4-nitro blue tetrazolium chloride (NBT), 5,5-dithio-bis-(2-nitrobenzoic acid) (DTNB), Hydrogen peroxide (H_2_O_2_), and Bovine serum albumin (BSA) were purchased from Sigma Aldrich (USA). 3-3′-Dihexyloxacarbocyanine iodide (DiOC6, Molecular Probes) and 2′,7′-dichlorofluorescein diacetate (DCFH-DA) were procured from Thermo Fisher Scientific (USA). All the cell culture media, buffer, and other reagents were obtained from Gibco (USA), and all other reagents used for this study were of the highest quality grade.

### 2.2 Preparation of HLD with MSG

Edible coconut oil and vanaspati ghee were collected from the market, and a mixture of the two was made in a 2:3 v/v ratio [3]. For the duration of the study, the animals were fed a high-fat meal at a dose of 10 mL/kg body weight orally daily in addition to the regular diet. The solution of MSG was prepared by dissolving 1.2 g of MSG in 20 mL of distilled water (600 mg/kg body weight) [1,4].

### 2.3 Experimental animals and plan of the work

Male adult albino Wistar rats (3 months old with body weights of 110 ± 10 g) were randomly selected for the present experimental setup. Rats were given a 7-day acclimatization period in the experimental animal house prior to the start of the experiment. The animals were housed in polypropylene cages with free access to water under conventional laboratory conditions of temperature (25 ± 2°C) and humidity (55 ± 5%), as well as a 12-hour light-dark cycle schedule. The rats were fed standard rat pellets. Throughout the trial, all the rats were given free access to food and water *ad libitum*. Cleaning and removal of feces and spilled feed from the cages on a daily basis ensured good hygiene. Rats were fed a regular meal consisting of 71% carbohydrate, 18% protein, 7% fat, and 4% salt mixture, as well as free access to water *ad libitum* [4].

Male albino rats of Wistar strain (110 ± 10 g) were used for the experiment. The experiment was carried out for 4 weeks. For the experiment, 24 healthy male adult albino rats of Wistar strain were randomly selected and were divided into 3 equal groups (*n* = 8) and treated as: Control (received normal saline 10 mL/kg body weight per day, orally), MSG (600 mg/kg body weight per day, orally) mixed with HLD (10 mL/kg body weight per day, orally), and MSG alone (600 mg/kg body weight per day, orally). The dose of HLD and MSG were selected as per our earlier studies [1,3,4]. Average body weight, organ weight, and spleen index [14] were calculated at the end of the treatment period (on day 29) just before sacrifice.


**Ethical approval:** The research related to animal use has been complied with all the relevant national regulations and institutional policies for the care and use of animals and was approved and performed according to the ethical instructions suggested by Serampore College Institutional Animal Ethics Committee, West Bengal, India (Project approval no. 25/P/S/SC/IAEC/2019) registered under Committee for the Purpose of Control And Supervision of Experiments on Animals (CPCSEA), Government of India (Reg. No. 1946/PO/Re/S/17/CPCSEA).

### 2.4 Determination of cytokine level

The levels of TNF-α and IL-6 were determined in a 96-well microplate by a sandwich enzyme-linked immunosorbent assay (ELISA) technique using an ELISA kit from Raybiotech, USA.

### 2.5 Method of isolation of splenocytes and separation of lymphocytes

The animals in all groups were anesthetized with an intraperitoneal injection of ketamine (87 mg/kg body weight) and sacrificed after 24h of fasting after the last application (29th day) [4]. Spleens from the individual animal were taken out and washed with ice-cold phosphate buffer saline (PBS). Spleens were cut into pieces and put in the ice-cold Alsever’s solution. Then, the spleen pieces were macerated with frosted glass slides in the Alsever’s solution in a 90 mm plastic Petri dish. Next, the suspensions were aspirated repeatedly to ensure single-cell suspension. The single-cell suspension was put into a lymphocyte separation medium that was taken in a centrifuge tube early. Then, it was centrifuged for 10 min at 2,500 rpm. After that, the buffy layer from it was taken out in a 10% RPMI-FBS solution [15].

### 2.6 Flow cytometric analysis of intracellular reactive oxygen species (iROS)

DCFH-DA was used to quantify iROS using BD FACSVerse with excitation at 488 nm, as directed by the manufacturer (Thermo Fisher Scientific, Waltham, MA, USA) (blue filter). Finally, data were evaluated using FlowJo software after the emission was collected using a 527/32 nm bandpass filter [3,4].

### 2.7 Analysis of mitochondrial transmembrane potential (MMP) by flow cytometry

MMP was assessed using the lipophilic dye DiOC6 (Thermo Fisher Scientific, Waltham, MA, USA) and analyzed using the BD FACSVerse at 488 nm, according to the instructions handbook (Thermo Fisher Scientific, Waltham, MA, USA). Finally, emission was measured using a 527/32 nm bandpass filter, and the results were analyzed with the FlowJo program [3,4].

### 2.8 Preparation of tissue extract

With a protease inhibitor cocktail, the spleen tissue homogenate was produced according to an earlier approach for determining nonenzymatic antioxidant, enzymatic antioxidant, and oxidative stress associated parameters [4].

### 2.9 Estimation of TBA reactive substances (TBARS) and nitric oxide (NO) production

TBARS generation was evaluated by TBA as a byproduct of lipid peroxidation [16]. The molar extinction coefficient (1.56 × 10^5^ cm^2^/mM) was used to convert the data into nmoles of TBARS per milligram of protein. Griess reaction was used to assess NO production [17]. The presence of nitrite in the sample was calculated using a sodium nitrite standard curve and represented in mol/mg of protein.

### 2.10 Estimation of SOD

The NBT method was used to calculate SOD activity based on the amount of time it takes for NBT to decrease [18]. The relative absorbance was then converted into units of SOD activity/mg of protein, with one unit of SOD activity equaling the quantity of SOD that caused a half-reduction in the NBT background rate.

### 2.11 Estimation of catalase (CAT)

The degradation of H_2_O_2_ at 240 nm was used to determine CAT activity using Beer’s protocol. The difference in absorbance per unit of time was used to estimate CAT activity [19]. The results were expressed as U/mg of protein.

### 2.12 Estimation of glutathione (GSH)

DTNB was utilized to estimate the level of GSH using the Ellman method. At 412 nm, the absorbance of reduced chromogen was measured spectrophotometrically. The amount of GSH in the body was calculated using a standard curve, and the results were represented in nmoles/mg of protein [20].

### 2.13 Estimation of tissue protein content

According to Lowry et al., BSA was used as a standard to calculate the protein in the homogenate of spleen tissue [21].

### 2.14 Histopathological examinations

Spleen was collected from all groups of animals and fixed in a 10% formaldehyde buffer solution before being processed. Routine microscope slides were prepared for hematoxylin and eosin (H and E) staining as per the standard method [3,4]. Stained slides were viewed under a light microscope for any alterations, and a photomicrograph was taken using a camera-attached compound microscope (Primo star model, Carl Zeiss Meditec, Dublin, CA).

### 2.15 Statistical analysis

To determine whether or not scores of different groups differ substantially and to assess significant intergroup differences, the Kruskal–Wallis non-parametric one-way analysis of variance (ANOVA) test and Mann–Whitney *U* multiple comparison tests, using StatsDirect 3.0 software (United Kingdom) were used. *P* < 0.05 was used to determine whether differences were significant [1,4].

## 3 Results

**Figure 1:**
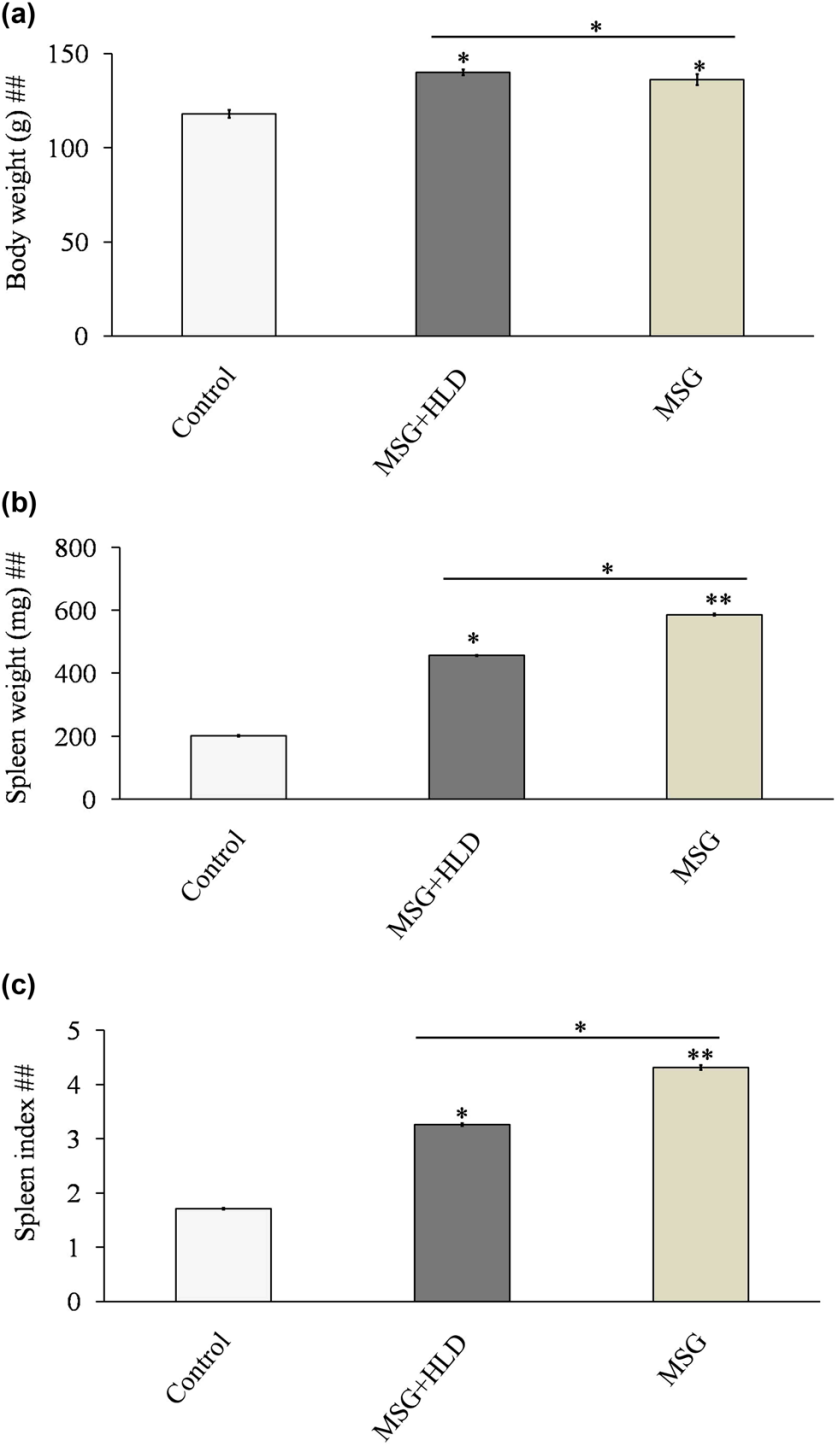
MSG with or without HLD induced changes in body weight (a), spleen weight (b), and spleen index (c). Significance level based on Kruskal–Wallis test [##*P* < 0.01]. Control vs MSG and Control vs MSG + HLD: **P < 0.01 and *P < 0.05.

The current investigation evaluates the adverse effect of MSG with or without HLD in the male rat model. In the present investigation, it can be stated that MSG is potentially more injurious than HLD in the spleen. MSG with or without HLD induces a significant increase in body weight (Control vs MSG + HLD, Control vs MSG, and MSG + HLD vs MSG: *P* < 0.05) as compared to the Control (Figure 1a). Furthermore, spleen weight also increases significantly (*P* < 0.01) in MSG-fed rats as compared to the Control (Control vs MSG + HLD: *P* < 0.05; Control vs MSG: *P* < 0.01; and MSG + HLD vs MSG: *P* < 0.05). MSG + HLD-fed animals also showed a similar type of increase in spleen weight as compared to the control (*P* < 0.05), but not equal to the MSG-fed group (Figure 1b). In addition, higher (a 1.91-fold increase in MSG + HLD group and a 2.52-fold increase in MSG group) spleen index (Figure 1c) in MSG + HLD and MSG-fed rats (Control vs MSG + HLD: *P* < 0.05; Control vs MSG: *P* < 0.01; and MSG + HLD vs MSG: *P* < 0.05) indicates severe infection in the spleen tissue which well corroborated with pro-inflammatory marker and oxidative stress-related parameters in the later section of the study. Therefore, it can be inferred that MSG with or without HLD causes splenomegaly, but the detrimental impact was higher in the MSG-fed group alone compared to the rest of the group.

### 3.1 Inflammatory response induced by MSG with or without HLD

**Figure 2:**
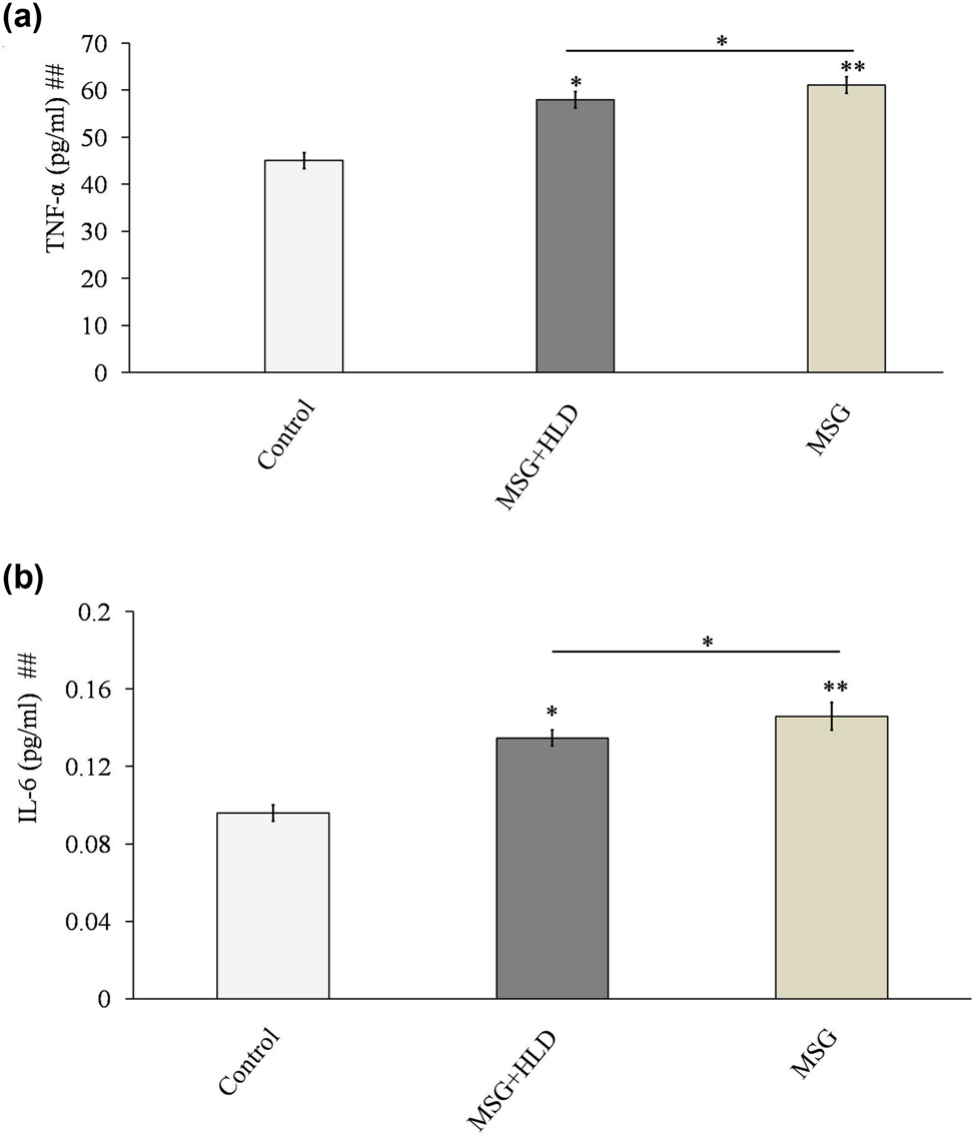
MSG with or without HLD induced changes in TNF-α (a) and IL-6 (b). Significance level based on Kruskal–Wallis test [##*P* < 0.01]. Control vs MSG and Control vs MSG + HLD: **P < 0.01 and *P < 0.05.

The present experimental setup indicates that MSG along with HLD causes inflammatory response (Figure 2) in MSG + HLD group (Control vs MSG + HLD: *P* < 0.05) via increasing pro-inflammatory IL-6 and TNF-α (IL-6 increased by 40.42% and TNF-α increased by 28.57%); although the higher production of such cytokines (IL-6 increased by 52.08% and TNF-α increased by 35.63%) were found in MSG group alone (Control vs MSG: *P* < 0.01). However, pro-inflammatory cytokines levels in the MSG + HLD- and MSG-fed rats also differ significantly (MSG + HLD vs MSG: *P* < 0.05). Thus, it can be stated that MSG induces a higher inflammatory response than MSG + HLD.

### 3.2 MSG with or without HLD generates ROS to cause oxidative stress

**Figure 3:**
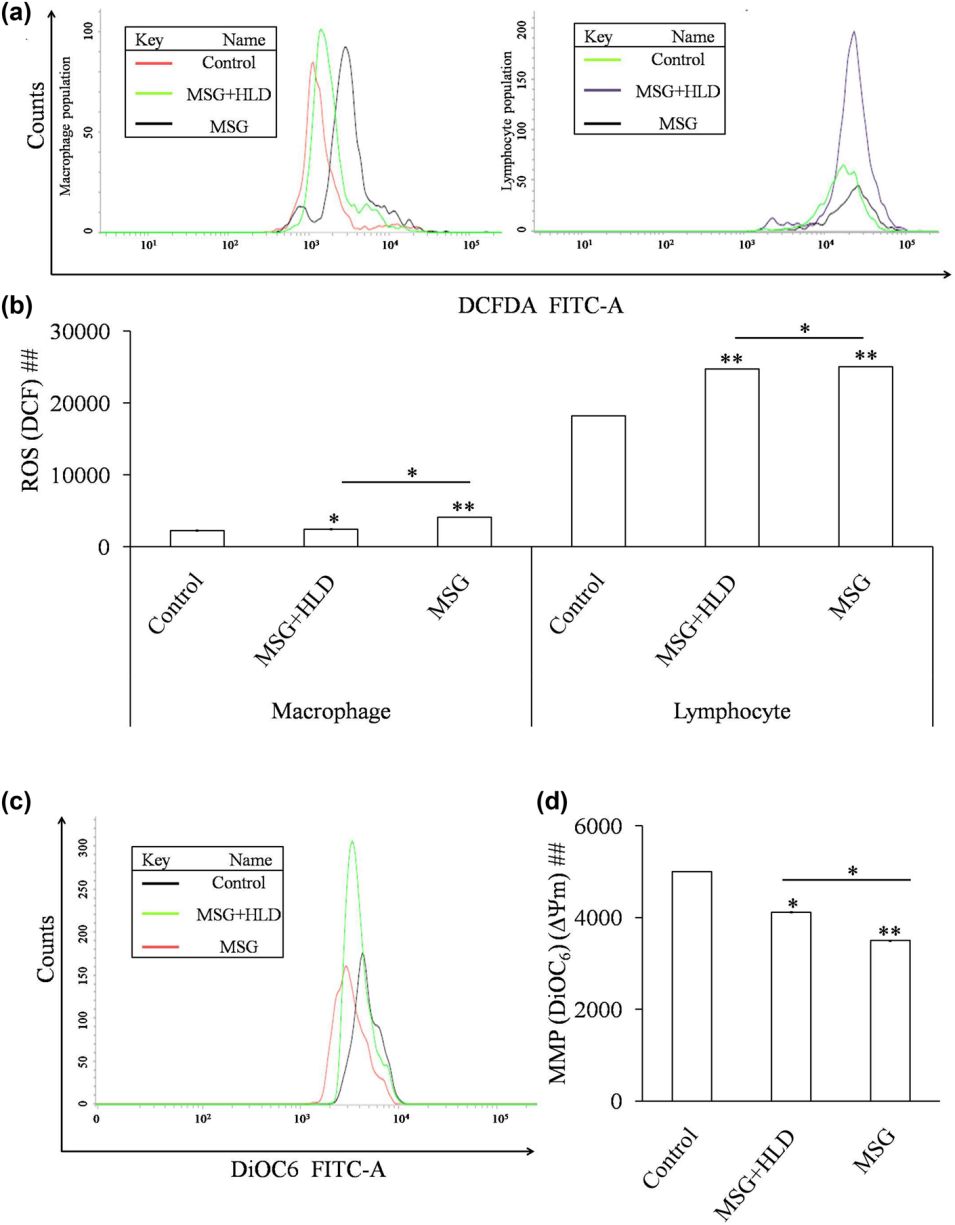
MSG with or without HLD induced oxidative stress via generation of iROS in macrophage population and lymphocyte population (a and b) and loss of MMP (c and d). Significance level based on Kruskal–Wallis test [##*P* < 0.01]. Control vs MSG and Control vs MSG + HLD: **P < 0.01 and *P < 0.05.

**Figure 4:**
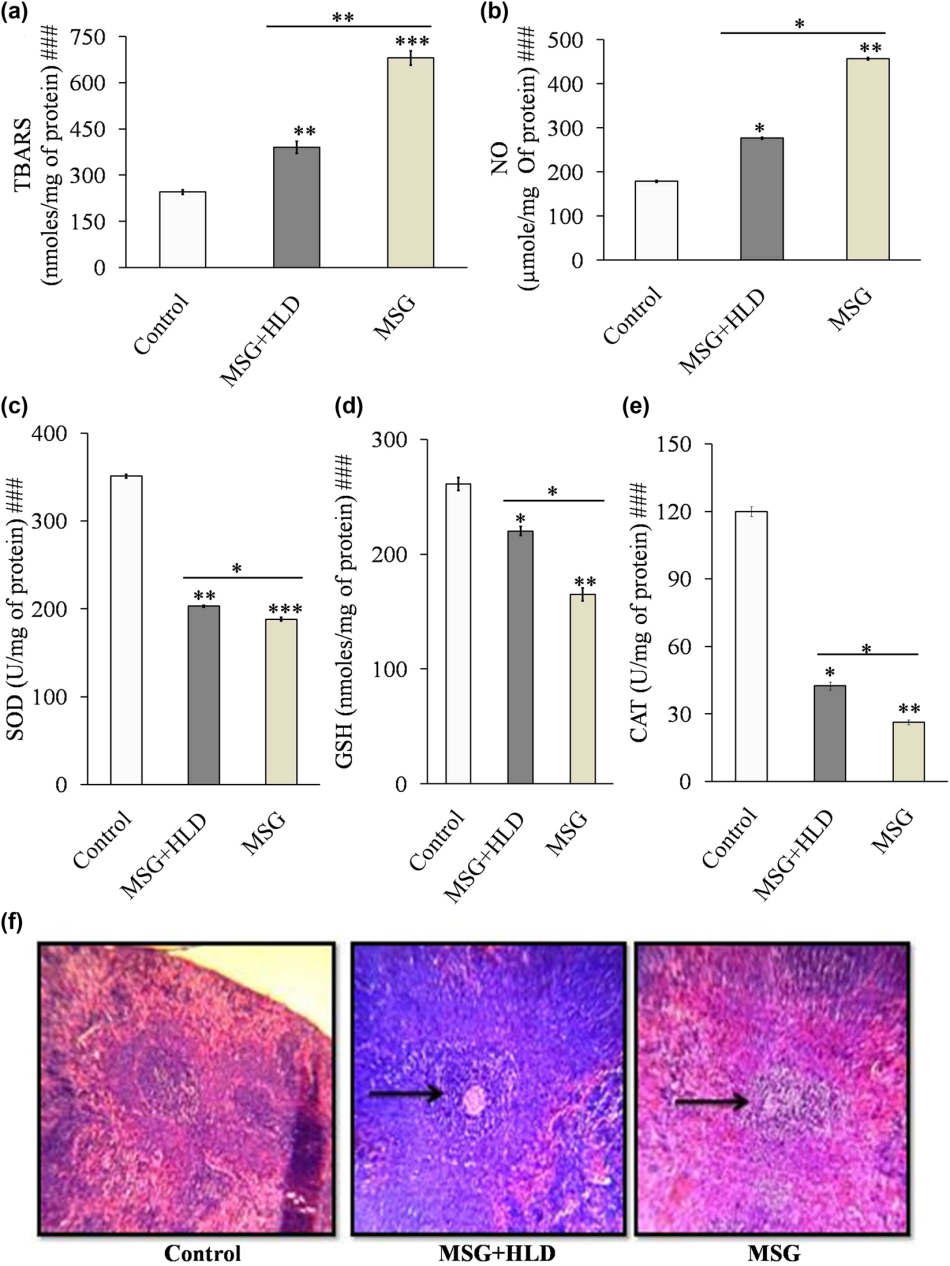
MSG with or without HLD induced changes in lipid peroxidation (a), NO (b), SOD (c), GSH (d), CAT (e), and spleen tissue architecture (f). Black pointed arrows indicate the degree of injury in spleen tissue by MSG or MSG + HLD. Significance level based on Kruskal–Wallis test [###*P* < 0.001]. Control vs MSG and Control vs MSG + HLD: ***P < 0.001, **P < 0.01 and *P < 0.05.

Inflammatory cytokines triggered the production of TBARS (Control vs MSG + HLD: *P* < 0.01 and Control vs MSG: *P* < 0.001) as a byproduct of lipid peroxidation and production of NO (Control vs MSG + HLD, *P* < 0.05 and Control vs MSG: *P* < 0.01) in spleen tissue homogenate in both MSG (TBARS increased by 177.51% and NO increased by 155.90%) and MSG + HLD-fed rats (TBARS increased by 59.30% and NO increased by 54.85%) as compared to the control (Figure 4a and b). In anticipation, GSH level (Control vs MSG + HLD: *P* < 0.05 and Control vs MSG: *P* < 0.01) and activities of antioxidant enzymes, particularly CAT (Control vs MSG + HLD: *P* < 0.05 and Control vs MSG: *P* < 0.01) and SOD (Control vs MSG + HLD: *P* < 0.01 and Control vs MSG: *P* < 0.001) were also decreased significantly (in case of MSG-fed group, GSH increased by 36.85%, CAT increased by 78.09%, and SOD increased by 46.42% and in the case of the MSG + HLD-treated group, GSH increased by 15.67%, CAT increased by 64.60%, and SOD increased by 42.21% ) in rats simultaneously treated with MSG and MSG + HLD (Figure 4c–e). Moreover, MSG and MSG + HLD groups also differ significantly (TBARS: *P* < 0.01; NO, SOD, CAT, and GSH: *P* < 0.05). Based on these results, it can be revealed that MSG with or without HLD induced oxidative damage in the spleen, which was well-corroborated with the generation of iROS. MSG generates 81.31% increase of iROS in macrophage population, 37.83% increase in lymphocyte population (Control vs MSG: *P* < 0.01) and MSG + HLD induces generation of 8.10% increase of iROS in macrophage population (Control vs MSG + HLD: *P* < 0.05), 35.87% increase in lymphocyte population (Control vs MSG + HLD: *P* < 0.01), (Figure 3a and b). Further, the generation of iROS in both macrophage and lymphocyte populations in MSG and MSG + HLD-fed groups differed significantly (MSG + HLD vs MSG: *P* < 0.05), which inferred that MSG causes deleterious impact on oxidative damage via generation of iROS than MSG + HLD.

### 3.3 Alteration of MMP by MSG with or without HLD associated with disruption of tissue architecture of the spleen

We have also checked the mitochondrial transmembrane potential in splenocytes of MSG and MSG + HLD-treated animals. Mitochondrial membrane potential is decreased in MSG-treated group by 30% (Control vs MSG: *P* < 0.01), and the MSG + HLD-treated group showed a 17.61% decrease (Control vs MSG + HLD: *P* < 0.05) in MMP as compared to the Control (Figure 3c and d). Additionally, MSG and MSG + HLD groups differ significantly (MSG + HLD vs MSG: *P* < 0.05) by reducing the MMP in the splenocytes.

All these events ultimately disrupt the normal architecture of the spleen (Figure 4f), both in MSG and MSG + HLD-fed animals. Spleen tissue examined under a light microscope indicated that MSG-fed group had serious cellular disruption, degeneration, and atrophy of white pulp. A further moderate degree of disruption was also revealed in the case of MSG in combination with HLD treated animals compared to Control, with no such pathophysiological changes. Therefore, it can be demonstrated that MSG causes severe spleen derangement by the inflammatory response by activating pro-inflammatory cytokines, oxidative stress followed by the generation of ROS, and lowering the MMP to cause mitochondrial malfunction.

## 4 Discussion

The spleen is the largest peripheral lymphoid organ in the body, contributing to immunity, lymphocyte production, erythrocyte aging elimination, and blood storage, but is more prone to oxidative stress-mediated damages [11]. The present study showed that MSG combined with HLD causes severe oxidative damage in the spleen by altering redox-guided cellular signaling. MSG induces a higher spleen index and oxidative reaction by increasing TBARS with reducing GSH levels. Oxidative stress may be a plausible explanation for the altered physiological responses, particularly in immune cells [22]. Alteration of redox-equilibrium via increasing lipid peroxidation along with NO production and reducing SOD, CAT, and GSH levels ultimately lead to oxidative stress-mediated damage in the spleen. Immune suppression caused by chemicals in animals can produce pathological alterations in the spleen and oxidative stress and immune cell apoptosis [23]. Moreover, the spleen weighs beyond the reported normal reference ranges and becomes a significant predictor of possible immunotoxicity, presumably related to the suppression of cell proliferation and stimulation of lymphocyte apoptosis [24,25]. The present study revealed that MSG and MSG + HLD-fed groups had increased spleen weights relative to their body weights, indicating spleen infection [14]. Thus, a higher spleen index in MSG-fed animals compared to MSG in combination with HLD induces immunomodulation and infection, which were well supported with the rest of the altered parameters.

In physiological conditions, the generation of ROS could be rapidly detoxified by intracellular antioxidant enzymes. Thus, in the present study, increased ROS in splenocytes of MSG and MSG + HLD-treated animals promotes oxidative stress, further stimulated by higher production of nitric oxide and TBARS. Similar findings have been observed previously in research involving other organs [26]. The tissues may be subjected to lipid peroxidation as their antioxidant status declines in an attempt to restore their normal oxidative state. Furthermore, increased lipid peroxidation in this study could be associated with a rise in H_2_O_2_ concentration in splenocytes, an inducer of oxidative stress via the generation of ROS [27]. The Fenton reaction converts H_2_O_2_ to hydroxyl radicals in the presence of transition metal ions, which are incredibly reactive and potentially hazardous substances that can disrupt peripheral organs. H_2_O_2_ is scavenged by CAT in normal conditions, resulting in the formation of water and oxygen. Due to a decrease in these enzymes’ activity and generation of ROS, the conversion of H_2_O_2_ to harmful hydroxyl radicals is significantly increased, which may contribute to MSG-induced oxidative stress. The present study results indicate that MSG with or without HLD causes free radical-mediated damage to the spleen tissue due to a lack of intracellular antioxidant enzymes. Reduced SOD activity failed to keep free radicals in balance, and higher TBARS level was among them. These findings are consistent with aberrant responses to environmental stresses and immunotoxin induction [22]. Further research is needed to understand whether these aberrant physiological responses are linked to the general immunosuppressive effects of MSG consumption.

**Figure 5:**
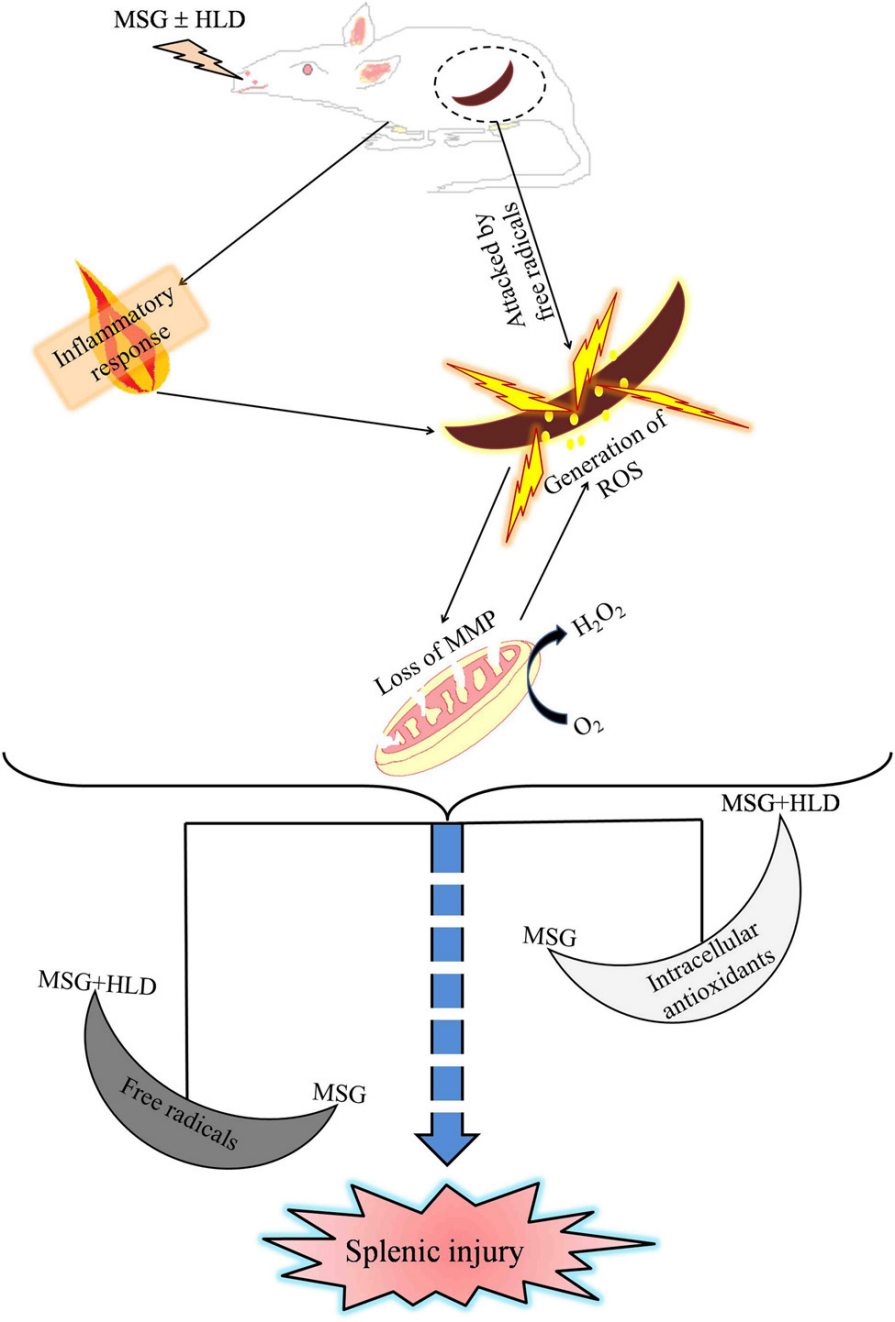
Hypothetical target pathway by which MSG with or without HLD causes spleen damage via altering redox status.

In addition, MSG has pro-oxidative properties as evidenced by excessive ROS formation, leading to the production of pro-inflammatory cytokines like TNF-α and IL-6. The present investigation showed that a significantly high level of TNF-α and IL-6 in MSG alone or in combination with HLD treatment promotes inflammatory response in the body, which further provokes splenocytes derangements, resulting in the spleen injury. On the other hand, mitochondria, the primary intracellular source of ROS, are vulnerable to oxidation but have a powerful antioxidant system [28,29]. The mitochondrial membrane permeability may rise in response to oxidative stress, with the generation of a voltage-dependent nonspecific pore in the inner membrane known as the mitochondrial permeability transition pore (MPTP) [30]. MPTP activation results in enormous enlargement of mitochondria, membrane depolarization, calcium release, rupture of the outer membrane, and the release of pro-apoptotic factors [31]. The present investigation uncovered that MSG and MSG + HLD altered the mitochondrial transmembrane potential as compared to control animals. The mitochondrial dysfunction is thought to be a crucial step in a ROS-mediated apoptotic pathway. Loss of mitochondrial membrane integrity in the spleen of MSG and MSG + HLD-treated animals indicates mitochondria-mediated programmed cell death [1,4]. Moreover, these events ultimately disrupt the spleen architecture by inducing oxidative stress along with inflammatory response via the formation of iROS and release of pro-inflammatory cytokines in MSG + HLD-fed rats, more prominently in MSG-fed group (Figure 5). However, MSG in combination with HLD also causes oxidative stress-mediated spleen injury, but the degree of damage is not up to the level exerted by MSG alone. In the present study, HLD has been prepared with coconut oil and vanaspati ghee in a ratio of 2:3; where vanaspati ghee is the source of hydrogenated fats and coconut oil contains saturated fatty acids like palmitic acid, myristic acid, lauric acid, and caprylic acid [3,4]. Apart from the hydrogenated fats and saturated fatty acids in HLD, coconut oil also contains polyphenols and medium chain triglycerides, which have antioxidant activity and a beneficial role in preventing metabolic syndrome, organ injury, and immunotoxicity [32,33,34,35]. Hence, it can be speculated that the degree of damage exerted by MSG is suppressed by HLD when the rats were treated with MSG + HLD as compared to the MSG alone. However, accumulating evidence suggested that MSG mixed in HLD or MSG alone cause nonalcoholic fatty liver disease (NAFLD) mediated systemic anomalies [1,4]; on the other side, another group of researchers found that the spleen plays a pivotal role in the development of NAFLD and steatohepatitis [36,37]. Hence, it can be speculated that MSG-induced splenic injury can further promote serious health issues like NAFLD. Therefore, it can be stated that MSG plays a dual role by modulating splenic homeostasis to cause the systemic anomaly.

## 5 Conclusion

In the present study, it can be concluded that treatment of MSG alone or in combination with HLD resulted in pronounced oxidative stress and cellular damage to the spleen by showing an inflammatory response. Furthermore, it can be speculated that MSG plays an immunosuppressive role when mixed with HLD to cause spleen damage. Further studies are required to find out the silent role of HLD on MSG-induced anomaly in the spleen.
